# Degradation Mechanisms in Metallized Barrier Films for Vacuum Insulation Panels Subjected to Flanging-Induced Stress

**DOI:** 10.3390/nano15161231

**Published:** 2025-08-12

**Authors:** Juan Wang, Ziling Wang, Delei Chen, Zhibin Pei, Jian Shen, Ningning Zhou

**Affiliations:** 1 School of Mechanical and Electrical Engineering, Hefei Technology College, Hefei 230012, China; 2 School of Energy, Materials and Chemical Engineering, Key Laboratory of Materials and Technologies for Advanced Batteries, Hefei University, Hefei 230601, China; 3 Materials Department, Advanced Research Center, Hefei Hualing Co., Ltd., Hefei 230000, China

**Keywords:** vacuum insulation panels (VIPs), barrier film degradation, flanging process, micro-crack propagation, metallized films

## Abstract

The long-term reliability of vacuum insulation panels (VIPs) is constrained by the barrier film degradation caused by micro-cracks during the flanging process. However, the correlation mechanism between process parameters and microleakage remains unclear. This study systematically investigates the impact of the number of flanging cycles on the barrier properties and insulation failure of aluminum foil composite film (AF) and metallized polyester film (MF). Accelerated aging tests revealed that the water vapor transmission rate (WVTR) of MF surged by 340% after five flanging cycles, while its oxygen transmission rate (OTR) increased by 22%. In contrast, AF exhibited significantly increased gas permeability due to brittle fracture of its aluminum layer. Thermal conductivity measurements demonstrated that VIPs subjected to ≥5 flanging cycles experienced a thermal conductivity increase of 5.22 mW/(m·K) after 30 days of aging, representing a 7.1-fold rise compared to unbent samples. MF primarily failed through interfacial delamination, whereas AF failed predominantly via aluminum layer fracture. This divergence stems from the substantial difference in mechanical properties between the metal and the polymer substrate. The study proposes optimizing the flanging process (≤3 bending cycles) and establishes a micro-crack propagation prediction model using X-ray computed tomography (CT). These findings provide crucial theoretical and technical foundations for enhancing VIP manufacturing precision and extending service life, holding significant practical value for energy-saving applications in construction and cryogenic fields.

## 1. Introduction

Vacuum insulation panels (VIPs) represent a transformative advancement in thermal management technology, delivering ultra-low thermal conductivity (1–4 mW/(m·K)) through synergized vacuum insulation and radiative shielding mechanisms [1,2]. With growing demands for energy-efficient solutions in building envelopes, cryogenic storage, and smart packaging, VIPs have emerged as critical components capable of reducing energy consumption by 30–50% compared to conventional insulation materials [3,4]. At the nanoscale, their performance hinges on the delicate equilibrium between vacuum integrity maintained by advanced barrier films and minimized thermal bridging enabled by nanostructured interfaces [5,6,7,8,9]. However, the long-term reliability of these systems remains constrained by an unresolved paradox: the pursuit of enhanced gas barrier properties through metallic layers inherently introduces thermal conduction pathways while creating mechanical vulnerabilities during manufacturing [10,11,12].

The evolution of VIP barrier films exemplifies nanotechnology’s pivotal role in balancing conflicting material properties. Early designs employed micrometer-thick aluminum foil (6–12 μm) laminates, achieving exceptional oxygen transmission rates but suffering from pronounced thermal bridging due to aluminum’s high intrinsic conductivity [13,14]. This limitation spurred the development of nanometallized barrier films, where plasma-deposited aluminum coatings (30–100 nm) on polyethylene terephthalate (PET) substrates reduced metallic mass by 99.7% while maintaining moderate barrier performance [15,16,17]. Contemporary designs further integrate ethylene vinyl alcohol (EVOH) interlayers, leveraging their semi-crystalline nanostructure (<5 nm crystalline domains) to create tortuous diffusion paths for gas molecules [18,19,20]. Despite these advancements, industrial adoption remains hindered by manufacturing-induced defects—particularly microcracks generated during the critical flanging process [3,21]. These subvisible flaws create preferential pathways for gas permeation, accelerating vacuum degradation and increasing thermal conductivity by 500–700% within months of service.

Three fundamental challenges persist at the intersection of nanomaterials engineering and VIP manufacturing: First, the mechanical mismatch between nanoscale metallic coatings and polymeric substrates induces interfacial stress concentrations during cyclic bending, leading to delamination and brittle fracture [22,23,24]. Second, conventional quality control methods lack sensitivity to detect nanoscale defects propagating from manufacturing stresses, allowing latent failures to escape detection [15,25,26]. Third, existing models fail to correlate process parameters (e.g., bending cycles) with the nanomechanical degradation mechanisms governing barrier performance. This knowledge gap severely limits the rational design of damage-tolerant nanolaminates, particularly hindering the development of mass-producible and qualified vacuum insulation materials.

This study systematically investigates the critical interplay between flanging process parameters and microleakage formation in VIPs, addressing a persistent knowledge gap in thermal insulation technology. By analyzing the correlation between gas permeability and thermal conductivity, establishing the relationship between flanging processes and performance degradation, and characterizing morphological changes at different evolution stages through optical microscopy and X-ray computed tomography (CT) imaging, the findings provide actionable insights for enhancing manufacturing precision and barrier film design. By bridging the gap between process engineering and material science, this work contributes to the sustainable development of high-performance thermal insulation systems, ultimately supporting global energy conservation initiatives.

## 2. Experimental Section

### 2.1. Experimental Samples and Preparation

The core material demonstrated in our paper is commercially available glass fiber (sourced from market-available products). The adsorbent is commercially available conventional materials and obtained from Sanhe Huazhen New Materials Co., Ltd. (Sanhe, Hebei, China). The adsorbent (model: AD103-V10) is primarily composed of mesoporous calcium oxide (CaO) blended with small amounts of copper oxide (CuO) and manganese oxide (MnO_2_) as active components. The encapsulation system integrates two distinct film layers: an aluminum foil composite (AF) featuring a 10 μm-thick aluminum core laminated with PET for enhanced mechanical stability, and a metallized polymer film (MF) comprising up to three aluminum-coated PET layers. This configuration ensures structural integrity while maintaining standardized commercial material specifications throughout the experimental system.

**Figure 1:**
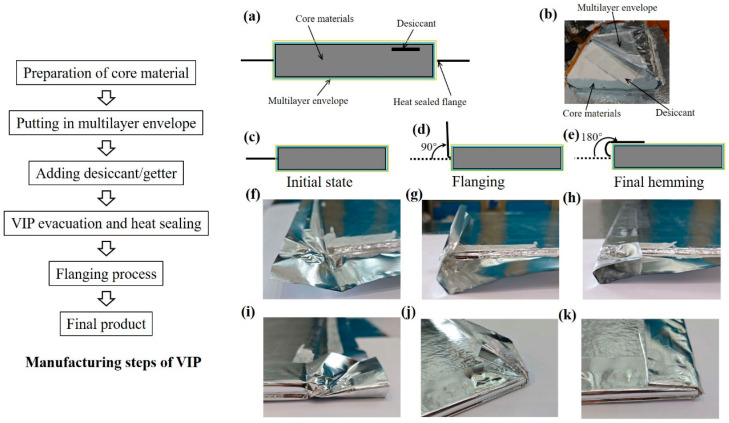
Schematic of the manufacturing process, including structure assembly, vacuum sealing, and post-processing. (**a**) Schematic and (**b**) real images of a VIP structure. Schematic illustration of the flanging process: (**c**) initial state, (**d**) flanging 90°, (**e**) final hemming state. Optical image of the flanging process: (**f**–**h**) correspond to the actual images of schematic (**c**–**e**), (**i**–**k**) are actual images of the adjacent-edge repetition process for (**c**–**e**).

The vacuum insulation matrix undergoes thermal-vacuum lamination manufacturing, with detailed fabrication steps shown in Figure 1. The VIP structure schematic is illustrated in Figure 1a. The main components consist of inner core materials, a multilayer encapsulation system, and desiccant/getters. The physical implementation (Figure 1b) shows preconditioned core materials sandwiched between gas-tight barrier films in a dual-layer encapsulation configuration, with integrated desiccant supplementation. Heat-sealing encapsulation is achieved through vacuum processing using an industrial-grade packaging system (XF2150, Zhaoqing Xinfeng Electromechanical Equipment Co., Ltd., Zhaoqing, Guangdong, China) featuring discrete evacuation and sealing modules. The multi-phase pumping assembly achieves base pressures below 5 × 10^−3^ Pa (unloaded state), preceding thermal sealing under stabilized vacuum (~6 × 10^−3^ Pa). This phase involves 160 °C plate compression for 15 s, exceeding the polyethylene sealant’s melting temperature (T_m_ ≈ 125 °C) to enable interfacial polymer coalescence, thereby creating permanent membrane-layer bonds. By applying heat and pressure, this technique permanently bonds barrier membranes to their encapsulating counterparts, achieving a gas-impermeable containment structure. Lastly, the post-encapsulation, edge-folding procedures are implemented (Figure 1c–e). Initially, peripheral envelope materials lie parallel to the core surface (Figure 1f). These are then folded upward at 90° (Figure 1g) and adhered onto the core-covering membrane (Figure 1h). This process is repeated on adjacent edges to establish cross-shaped overlapping regions (Figure 1i–k), ensuring structural integrity. VIP samples subjected to accelerated aging had dimensions of 600 mm × 400 mm × 10 mm. All samples were prepared under identical processing conditions. By repeatedly bending the folded edge positions 0, 1, 3, 5, and 10 times, the performance of the barrier bags and VIPs was tested.

### 2.2. Accelerated Aging Test

VIP samples (600 mm × 400 mm × 10 mm) subjected to 0, 1, 3, or 5 bending cycles were placed in a high-temperature accelerated aging chamber (70 °C, 65% RH) for 30 days. A single folding cycle refers to one complete sequence of (1) mechanically folding the sample to a predefined angle (e.g., 180°) along the designated crease line; (2) fully returning the sample to its original flat configuration (unfolding). Each fold/unfold action was performed smoothly and consistently within 5 s to ensure uniformity across cycles. All bending treatments were completed prior to aging initiation. Samples were periodically retrieved from the aging chamber at predetermined intervals (Days 0, 7, 15, 21, 30) under controlled experimental protocols. Following extraction, samples were immediately transferred to a controlled testing environment (25 °C) for a 24 h thermal equilibration period. Thermal conductivity measurements were conducted within two hours of equilibration completion. To maintain temporal accuracy in aging exposure, all specimens were systematically returned to the chamber within 30 min post-measurement. Notably, measurement intervals were excluded from cumulative aging duration calculations.

### 2.3. Oxygen Transmission Rate (OTR)

OTR measurements were conducted under strictly maintained environmental controls (23 ± 0.5 °C, 0% RH). The experimental setup incorporated a C203E isostatic differential oxygen transmission tester (Labthink Instruments Co., Ltd., Jinan, Shandong, China) utilizing coulometric sensing with pressure equilibration. A dual-compartment configuration separated by the test specimen was implemented: the upper section pressurized with high-purity oxygen (99.995% certified grade, 101.3 kPa), contrasting with the lower chamber purged with ultra-high-purity nitrogen (99.999%, Hefei Shenhe Gas Co., Ltd., Hefei, Anhui, China). Permeating oxygen molecules were convectively delivered through the N_2_ carrier stream to a high-sensitivity electrochemical detector (resolution: 1 × 10^−4^ cm^3^/(m^2^·day)). Standardized circular specimens (50 cm^2^ effective test area) were die-cut prior to analysis, with triplicate experimental runs conducted per sample and results expressed as mean values.

### 2.4. Water Vapor Transmission Rate (WVTR)

WVTRs were measured using a modulated infrared absorbance detection instrument (C303E, Labthink Instruments Co., Ltd., Jinan, Shandong, China). Tests occurred in a controlled environment (38.0 ± 0.2 °C, 90 ± 2% RH) within a dual-chamber permeation cell. This cell provided a 50 cm^2^ test area for circular specimens (diameter: 7.98 cm, area = π × (3.99 cm)^2^) and maintained chamber RH automatically (± 0.5% accuracy). The upper chamber received humidified synthetic air (90% RH), contrasting with the lower chamber’s dry N_2_ flow (99.999% purity, 10 cm^3^/min), which carried permeated water molecules to a wavelength-modulated IR sensor (0.01 g/(m^2^·day) detection limit). Circular specimens (50 cm^2^ effective area) were prepared by die-cutting. To mimic packaging use, specimens were placed with the PA layer oriented towards the humidified chamber. Six consecutive measurements per sample were performed, incorporating automatic gas purge cycles between each run.

### 2.5. Optical Photos

Optical micrographs of the samples were captured via a transmission-based imaging system (WV-CP490/CH, Panasonic, Kadoma, Osaka, Japan). Rectangular sections measuring 1.5 mm × 20 mm were prepared for cross-sectional analysis. Emphasis was placed on assessing crease morphology within predefined folding regions subjected to 1, 3, and 5 cycles of folding.

### 2.6. Thermal Conductivity Measurement

The thermal conductivity of the VIP was determined using a FOX 600 GHP Heat Flow Meter (TA Instruments, New Castle, DE, USA) operating on the steady-state heat flux principle. The sample was positioned between thermally regulated plates set at 10 °C (cold side) and 40 °C (hot side), maintaining a mean temperature approximating ambient conditions. A stable unidirectional steady-state heat flux was established across both the instrument’s central measurement region and the VIP core. During testing, the system ensured precise temperature control and consistent thermal gradients between the plates. Heat flux through the sample was quantified, enabling calculation of the thermal resistance ratio between the VIP and a calibrated reference material.

Under steady-state conditions, the heat flux (*q*) through the sample was calculated using Fourier’s Law:

(1) $$q=-k\times \frac{∆T}{d}$$

where *k* is the thermal conductivity of the sample (W/(m·K)). Δ*T* is the temperature difference across the sample (K), measured by embedded thermocouples. *d* is the thickness of the sample (m).

The thermal resistance (*R*) of the VIP and reference material was determined as:

(2) $$R_{VIP}=\frac{∆T_{VIP}}{q\times A}$$

(3) $$R_{Ref}=\frac{∆T_{Ref}}{q\times A}$$

where *A* is the cross-sectional area (m^2^).

The thermal resistance ratio (*η*) is then:

(4) $$\eta =\frac{R_{VIP}}{R_{Ref}}$$

The reference material was pre-calibrated using a guarded hot plate apparatus to validate its *k* value, ensuring traceable measurements.

### 2.7. X-Ray Computed Tomography (CT)

X-ray imaging leverages differential X-ray absorption across materials of varying composition or thickness to enable non-destructive internal structural analysis and defect characterization. This method provides real-time, high-resolution visualization for identifying the presence, classification, and severity of internal flaws. Analyses were conducted using an ISD-NI-RX85 offline high-precision X-ray non-destructive testing system (Hangzhou Ruiying Technology Co., Ltd., Hangzhou, Zhejiang, China), which integrates seven core components: (1) a micro-focus X-ray source, (2) an image acquisition unit, (3) a computer-based image processing module, (4) a precision mechanical system, (5) an automated electrical control unit, (6) radiation safety shielding, and (7) an alarm monitoring subsystem. The testing parameters were set to 35 kV voltage and 30 μA current under optimized conditions.

## 3. Results and Discussion

**Figure 2:**
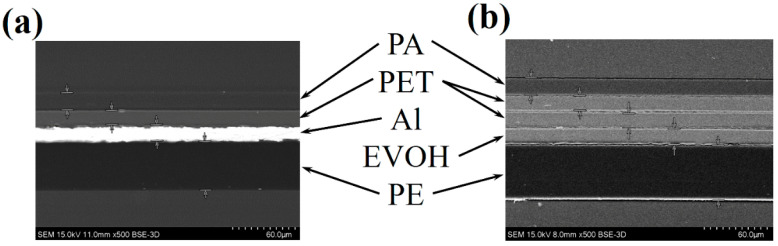
Cross-section images of (**a**) laminated aluminum foil (AF), and (**b**) multilayer membrane with metallized films (MFs).

The barrier film comprises three functional layers: a structural layer, a heat-sealing encapsulation layer, and a gas barrier. The structural layer ensures mechanical integrity, while the innermost heat-sealing layer facilitates hermetic sealing. The gas barrier layer suppresses the air and water vapor permeation, thereby extending VIP service lifetimes [7]. As illustrated in Figure 2, VIP envelopes employ two multilayer membrane architectures designed to synergize material properties for enhanced performance. Figure 2a depicts a conventional barrier film consisting of a 15 μm polyamide (PA) protective outer layer, a 14 μm PET intermediate layer, a 15 μm aluminum (Al) foil layer, and a 50 μm polyethylene (PE) heat-sealing layer. The Al film exhibits superior barrier properties with minimal water vapor and oxygen transmission rates. However, such metallic components induce inherent thermal bridging, elevating thermal conductance relative to non-metallic alternatives [5]. To reconcile insulation efficiency with barrier performance, nanoscale metallized multilayers have emerged as advanced VIP solutions. Modern designs utilize plasma-enhanced chemical vapor deposition (PECVD) to apply ultrathin aluminum coatings (30–100 nm) onto flexible 12–25 μm PET substrates (Figure 2b). However, intrinsic microdefects in metallized layers permit gas permeation proportional to both pinhole density and aluminum thickness [27]. This limitation is addressed through EVOH integration, which compensates for potential defects or inhomogeneities in the aluminum coating via its molecular-level crystalline barrier structure. This combination ensures enhanced overall performance compared to either material alone. Both EVOH and PET are thermoplastic polymers, enabling compatibility in co-extrusion or lamination processes. Their similar thermal and mechanical behaviors allow seamless integration into multilayer films, ensuring scalability and industrial feasibility. The optimized configuration reduces metallic mass by 99.7% while maintaining barrier efficacy and lowering thermal conductivity by 40–60% through minimized interfacial heat transfer [28,29].

**Figure 3:**
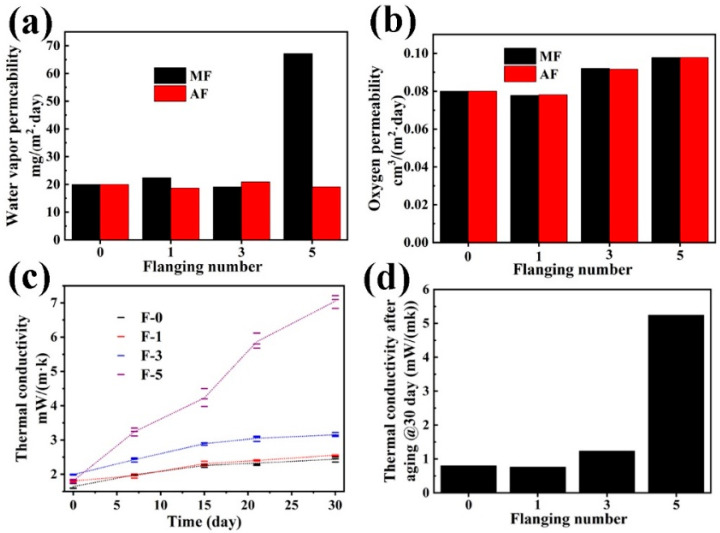
(**a**) Water vapor transmission rate, (**b**) oxygen gas transmission rate of different flanging numbers between AF and MF samples. (**c**) Variation in thermal conductivity with time and (**d**) increment of thermal conductivity after aging 30 days at 70 °C/65% RH of different flanging number VIP samples. Bending cycles (n = 0, 1, 3, 5) are denoted as F-0, F-1, F-3, F-5.

The performance of these barrier systems is ultimately validated through gas permeation dynamics. VIP envelopes primarily preserve vacuum integrity by inhibiting water vapor and gaseous permeation, with transmission occurring via intrinsic material permeability or interfacial leakage at sealant junctions [12]. Comparative analysis of post-deformation barrier membranes revealed divergent permeation characteristics. AF membranes demonstrated stable WVTRs during cyclic bending stress (19.5 mg initial vs. 20 mg after five cycles), whereas MF membranes exhibited significant WVTR degradation from 20 mg to 68 mg (3.4-fold increase) upon the fifth bending cycle (Figure 3a). Both membrane types manifested progressive OTR deterioration, recording respective 15% and 22% increases after three and five bending cycles, respectively, thereby confirming mechanically induced barrier property degradation (Figure 3b). The observed slight decrease in OTR (from 0.08 to 0.078 cm^3^/(m^2^·day)) after the first bending cycle is likely attributed to minor test variability within the measurement precision of the equipment. This small deviation may not reflect a true material property change but rather inherent experimental uncertainty. These observations highlight the critical relationship between deformation-induced structural modifications and gas barrier efficacy in VIP systems. Thermal conductivity measurements of VIP specimens subjected to sequential bending cycles (0, 1, 3, 5) after thermal aging revealed distinctive patterns (Figure 3c). No significant variation in initial thermal conductivity was observed, even after five bending cycles. After 10 bending cycles, the samples developed macroscopic fractures in the membrane layer, resulting in failure to maintain vacuum integrity. The measured thermal conductivity (25.2 and 33.2 mW/(m·K)) matched that of the core material, confirming the complete loss of high insulation efficiency from the vacuum. During accelerated aging protocols, singly bent VIPs maintained thermal conductivity trajectories comparable to control specimens. While panels subjected to ≥3 bending cycles demonstrated significant thermal conductivity escalation over time, confirming barrier film compromise. Quantitative analysis revealed a bending-dependent degradation profile, with 30 day aged specimens showing thermal conductivity increases of 1.12 mW/(m·K) (three cycles) and 5.22 mW/(m·K) (five cycles), with values 1.5–7.1 times greater than control (0.77 mW/(m·K)) and single-cycle specimens (0.74 mW/(m·K)) (Figure 3d). The minor difference in thermal conductivity between the control and single-cycle specimens is likely within the range of experimental uncertainty. The closeness of these values (a difference of ~4%) suggests that a single bending cycle does not induce significant structural damage to the material. This progressive deterioration pattern strongly suggests that cyclic mechanical stress induces microstructural defects in barrier membranes, thereby accelerating gaseous permeation kinetics and potentiating vacuum degradation mechanisms.

**Figure 4:**
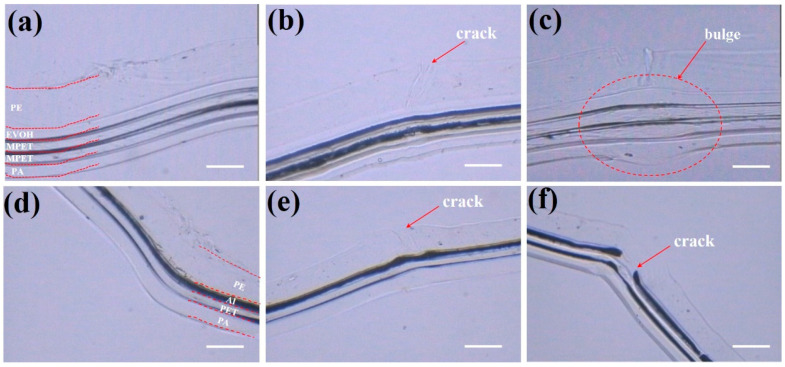
Cross-sectional optical micrographs of the different flanging numbers between AF and MF samples. (**a**) 1, (**b**) 3, and (**c**) 5 cycles for MF, (**d**) 1, (**e**) 3, and (**f**) 5 cycles for AF. The scale bar in the image is 50 μm.

The observed degradation in thermal conductivity and gas barrier performance indicates that repeated bending compromises the integrity of the barrier film, accelerating gas permeation and contributing to vacuum failure. To investigate the underlying microscopic mechanisms responsible for this deterioration, this study systematically analyzes the evolution of cross-sectional morphology under varying bending cycles (n = 1, 3, 5) using optical microscopy. The results demonstrate a clear correlation between the formation of micro-cracks and the development of microleakage pathways. Figure 4a illustrates the well-defined five-layer architecture of MFs, consistent with the structural schematic representation in Figure 2b. Following three bending cycles, the overall structural integrity is maintained, but distinct cracking is induced in the PE layer (Figure 4b). The overall thickness of the material increased significantly from ~112 μm to ~155 μm after five bending cycles (Figure 4c). This substantial thickening suggests volumetric expansion within the layered structure, which is attributed to interfacial delamination between layers. Such delamination could create localized voids or gaps that contribute to the observed thickness change, even if not directly visible in the cross-sectional image. The blister-like features at the fold lines exhibit a bidirectional bulge rather than a unidirectional deformation. This symmetric expansion is inconsistent with simple plastic deformation or buckling, which typically produces curvature in a single direction. Instead, it strongly implies interfacial separation, where adjacent layers detach and displace outward, forming internal pores that manifest as surface bulges. These observed mechanical failures correlate directly with the measured 340% increase in WVTR and the 22% enhancement in oxygen permeability demonstrated in Figure 3a,b. Progressive delamination of the EVOH interlayer under repeated stress, creating tortuous pathways for water vapor permeation. Pinhole coalescence in the nanoscale Al coating, which amplifies permeation exponentially once interconnected cracks form (critical threshold reached at cycle 5). In contrast, AFs exhibited differential failure mechanisms despite sharing a similar four-layer construction (Figure 4d). While comparable PE layer fracturing occurred after three cycles (Figure 4e), advancing to five bending cycles provoked complete fracture of the aluminum metallization layer (Figure 4f). This observed aluminum layer fragmentation, which conflicts with bulk aluminum’s inherent ductility, arises from two synergistic mechanisms inherent to the laminated system. First, during edge folding, the asymmetric mechanical constraints imposed by the 50 μm-thick PE layer and 30 μm-thick PA/PET composite layer subject the 15 μm-thick aluminum foil to localized compressive stresses at bending radii approaching 180° (Figure 1). These constraints exceed the local ductility limit of the metallization layer, initiating fracture nucleation. Second, cyclic bending induces fatigue-driven damage accumulation, with interfacial strain concentrations progressively weakening the aluminum layer until catastrophic crack propagation and interfacial delamination occur. While bulk aluminum demonstrates excellent macroscopic ductility, the confined geometry and multilayer interactions in this system generate unique stress states that override the material’s intrinsic plasticity, ultimately compromising both thermal conduction and barrier functionality.

**Figure 5:**
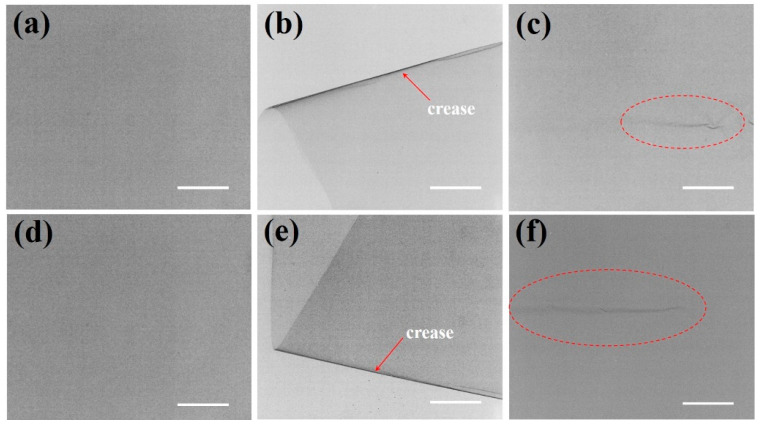
X-ray computed tomography images of different states between AF and MF samples. MF: (**a**) Initial state, (**b**) bending process, (**c**) final hemming state. AF: (**d**) Initial state, (**e**) bending process, (**f**) final hemming state. The scale bar in the image is 5 mm.

The observed mechanical degradation originates from mismatched deformation behaviors between metallic and polymeric constituents in the laminated system. This system utilizes polyurethane adhesive bonding, where the polymer’s inherent flexibility initially preserves structural coherence. However, cyclic bending stresses progressively compromise interfacial adhesion, inducing localized delamination that substantially elevates gas permeability. Specifically, aluminum (Al) foil layers fail through strain-localized cracking due to their limited ductility under bending stresses (Figure 4f), while metallized polymer films degrade via interfacial delamination at metal/polymer interfaces (Figure 4c), creating permeation pathways [21,25]. Although PE layer fractures are observed at fold edges (Figure 4b,c,e), these localized polymer failures primarily reflect stress concentration effects rather than governing barrier degradation, which is dictated by metallic component integrity and interfacial adhesion stability. Critically, thermal conductivity measurements demonstrated significant deterioration after only three bending cycles, despite negligible alterations in surface morphology. This suggests the development of subsurface micro-cracks within the metallic layer. The CT imaging technique, which is highly sensitive to density contrasts, reveals crack-like discontinuities localized within the Al layer. The significant difference in X-ray attenuation between the Al layer (high attenuation) and polymer layers (low attenuation) allows CT to resolve subsurface cracks specifically within the metal. CT analysis substantiated this hypothesis through differential X-ray attenuation contrast at material interfaces. Figure 5a,d are the unfolded membranes. The entire field of view in these panels corresponds to the pristine membrane without edge-folding treatment. No significant grayscale contrast differences are observed across the entire area. This conclusion is consistent with that reported in existing literature [26]. This uniformity in attenuation profiles indicates the absence of internal cracks or microstructural defects in the pristine specimens. In contrast, stress concentration during bending manifested as regions of increased attenuation contrast along the crease (Figure 5b,e). During bending, stress concentration occurs preferentially along the crease due to localized mechanical deformation. This stress concentration induces microstructural changes within the material, which significantly alter X-ray attenuation properties. Consequently, these regions exhibit enhanced contrast compared to the surrounding areas (labeled with red arrows in Figure 5b,e). Moreover, after a single edge-folding process, distinct grayscale gradients (Figure 5c,f) become evident. These localized variations in attenuation correspond to crack initiation and propagation within the membrane, confirming that mechanical deformation induces microstructural damage. Based on CT analysis, the subsurface cracks in the aluminum layer are estimated to have a width of ~100 μm and a length extending proportionally to the folded edge dimensions. These micro-cracks disrupt the continuous conductive pathways in the aluminum layer, leading to the observed rapid decline in thermal conductivity after only three bending cycles. Furthermore, fractures observed in the AF specimens exhibit greater length and intensity in attenuation contrast, indicating more extensive damage to the monolithic aluminum layer compared to the metallized polymer substrate, as shown by the red dotted circles in Figure 5c,f.

## 4. Conclusions

This study systematically examines the critical relationship between flanging-induced mechanical stresses (quantified via cycle counts of n = 0, 1, 3, 5) and microleakage formation in VIPs, establishing a quantitative framework that advances fundamental understanding of barrier film degradation mechanisms under cyclic mechanical loading. Experimental analyses demonstrate that repetitive bending cycles during flanging operations progressively compromise hybrid barrier film integrity, significantly degrading gas barrier performance and thermal insulation efficacy. For MFs, bending beyond three cycles induces interfacial delamination and localized blistering, resulting in a 340% increase in WVTR and 22% oxygen permeability deterioration. AF laminates exhibited brittle fracture within the metallic layer under equivalent bending stresses, creating enhanced thermal bridging pathways and gas permeation channels. Accelerated aging tests confirmed that VIPs subjected to ≥3 bending cycles experienced thermal conductivity increments up to 5.22 mW/(m·K), with this value representing a seven-fold rise compared to control specimens.

Combined optical microscopy and CT revealed distinct failure modes in VIP structures. MF membranes exhibited adhesive layer delamination and polymer matrix cracking, whereas AF structures underwent catastrophic metallic layer fragmentation. These divergent failure behaviors stem from the distinct mechanical characteristics of polymeric and metallic components, intricately manifested through their stress responses to cyclic folding. The quantified folding cycle thresholds (three cycles) empirically demonstrate this disparity, establishing critical connections between manufacturing parameters and material-specific failure modes. Specifically, the high elastic modulus of aluminum (68–72 GPa) predisposes AF structures to fragmentation under cyclic folding, occurring within <3 cycles. While bulk aluminum exhibits ductility, our constrained layer configuration (sandwiched between polymer films) induces localized strain concentrations that accelerate fatigue crack propagation, ultimately manifesting as brittle fracture patterns. In contrast, MF membranes demonstrate progressive interfacial failure through the folding cycles. These findings emphasize that interfacial adhesion strength and the metallic layer critically govern barrier durability. Future developments should focus on enhancing polymer–metal interlayer integrity through advanced adhesion techniques, coupled with in-process quality control via X-ray computed tomography. These improvements will enable the production of next-generation VIPs with extended service life, reduced thermal bridging, and enhanced reliability for energy-critical applications in construction and cryogenic systems.

## Data Availability

The original contributions presented in this study are included in the article. Further inquiries can be directed to the corresponding author(s).
